# Skin Lesion Detection Using Hand-Crafted and DL-Based Features Fusion and LSTM

**DOI:** 10.3390/diagnostics12122974

**Published:** 2022-11-28

**Authors:** Rabbia Mahum, Suliman Aladhadh

**Affiliations:** 1 Department of Computer Science, University of Engineering and Technology, Taxila, Taxila 47040, Pakistan; 2 Department of Information Technology, College of Computer, Qassim University, Buraydah 51452, Saudi Arabia

**Keywords:** deep learning, benign, malignant, skin cancer, skin lesion, HCI

## Abstract

The abnormal growth of cells in the skin causes two types of tumor: benign and malignant. Various methods, such as imaging and biopsies, are used by oncologists to assess the presence of skin cancer, but these are time-consuming and require extra human effort. However, some automated methods have been developed by researchers based on hand-crafted feature extraction from skin images. Nevertheless, these methods may fail to detect skin cancers at an early stage if they are tested on unseen data. Therefore, in this study, a novel and robust skin cancer detection model was proposed based on features fusion. First, our proposed model pre-processed the images using a GF filter to remove the noise. Second, the features were manually extracted by employing local binary patterns (LBP), and Inception V3 for automatic feature extraction. Aside from this, an Adam optimizer was utilized for the adjustments of learning rate. In the end, LSTM network was utilized on fused features for the classification of skin cancer into malignant and benign. Our proposed system employs the benefits of both ML- and DL-based algorithms. We utilized the skin lesion DermIS dataset, which is available on the Kaggle website and consists of 1000 images, out of which 500 belong to the benign class and 500 to the malignant class. The proposed methodology attained 99.4% accuracy, 98.7% precision, 98.66% recall, and a 98% F-score. We compared the performance of our features fusion-based method with existing segmentation-based and DL-based techniques. Additionally, we cross-validated the performance of our proposed model using 1000 images from International Skin Image Collection (ISIC), attaining 98.4% detection accuracy. The results show that our method provides significant results compared to existing techniques and outperforms them.

## 1. Introduction

Skin melanoma is a life-threatening disease that appears on areas of the body more exposed to sunlight. To analyze the abnormal growth of cells on skin, biomedical imaging plays a vital role. Moles are assessed through imaging techniques, which aid in the early detection and treatment of chronic diseases [1]. Cells and biological tissues are examined to investigate illness of the human organs. These organs are scanned with the help of medical imaging modalities such as X-ray, immunohistochemistry images, ECG, and MRI [2]. CT (SPECT), PET scan, X-ray and MRI are some of the medical imaging modalities that are = frequently utilized by medical experts and physicians. The physiological functions and biological structure of various organs can be analyzed by using these imaging modalities [3]. Moreover, numerous types of tumor, i.e., brain, blood, skin, and lungs are mostly analyzed and detected using CT scans. Dysplasia is clinically visualized with autofluorescence imaging (AFI), high-resolution endoscopy (HRE), narrow-band imaging (NBI), and confocal laser endo-microscopy (CLE) and optical coherence tomography (OCT) [4]. Tumor tissues, adipose tissues, and breast cancer can be easily diagnosed by X-ray mammography [5,6]. X-ray mammography is painful; therefore, advanced microwave imaging techniques are used for breast cancer detection [7]. Cardiovascular and dental health analysis is performed by CT [8,9]. These imaging modalities are used to diagnose the aforementioned diseases; however, we are concerned about how skin lesions can be detected using these biomedical imaging modalities.

Dark or red pigments on the skin are recognized as skin lesions [10]. These can be classified into primary and secondary types. Primary skin lesions are present on the body at birth. Secondary skin lesions are produced by abnormal cells growth. Skin lesions that are produced by the abnormal growth of cells are known as tumors or cancer. Alteration in normal cells and uncontrolled growth produces cancer in the body. Skin cancers are of two categories, i.e., melanoma and non-melanoma. Malignant melanoma is the most dangerous type of skin cancer and can cause death; however, it is 20 times less common than other type of skin cancer [11]. Annual cases of malignant melanoma have been increased in the last few decades, although it is uncommon for them to be caused by a change in lifestyle and consuming unhealthy food. Skin cancer occurrence continues to rise globally [12]. In India, more than 5000 patients are admitted with skin cancer every year, among which 4000 die [13]. Skin cancer diagnosis performed by clinical screening with the naked eye is difficult due to the heterogeneous appearance and irregular shape of tumors [14]. Four features are used for skin lesion analysis: mole diameter, color uniformity, asymmetry of the mole, and border irregularity of the mole. These features cannot be observed accurately with manual detection methods. Due to the increase in skin cancer, computer-aided methods are needed for accurate and timely detection, and dermatologic ultrasound is mostly used for the diagnosis of skin lesions [11]. However, skin cancer can also be diagnosed by histopathology analysis. Biopsy in histopathology is a painful process, which limits its usage in clinical practices [15]. Therefore, image analysis techniques are preferred due to their low cost and timely detection, such as laser scanning, MRI, ultrasound, and optical coherence tomography. Therefore, there is room for automated skin cancer detection that can identify cancer in its early stages.

Various techniques have been developed to detect the diseased portion of the skin due to melanoma. Several traditional machine learning-based techniques have been proposed such as [16,17]. However, these techniques do not provide significant results due to moles’ varying size, shape, and color. Moreover, the features were extracted manually, which is a tiring task and requires extra human effort. Some segmentation-based methods have also been developed, such as [18], using thresholding. The methods based on segmentation performed better than classical machine learning approaches. Moreover, these techniques are employed on the segmented part of the image named as the region of interest (ROI). The non-affected part of the skin is excluded in the segmentation-based method, as it may cause formation of the weak feature vector. Many other techniques based on segmentation have been developed due to their better detection results [19,20]. However, the ROI-based techniques provided better detection accuracies, but the thresholding methods performed significantly for non-varying contrast, illuminations, and chrominance. Consequently, deep learning-based techniques [21] were formed for automated skin cancer detection. These methods are able to compute the most valuable features from the affected part of the skin, considering variations such as illumination and intensity. They are capable of automatic features extraction and overcoming the challenges of localization and detection.

In this study, we propose a features fusion-based method for the early detection of skin cancer. The features were fused after extraction using a traditional machine learning-based and deep learning-based algorithm. The suggested model effectively detected and classified skin cancer due to its hybrid architecture. The model comprises three steps, namely pre-processing using Gaussian filtering (GF), features extraction (FE), and classification. The local binary patterns (LBP) algorithm was employed for hand-crafted features extraction. Then, DL-based features were extracted using the Inception v3 technique. To optimize the detection accuracy, a learning rate scheduler, i.e., Adam was employed in the proposed model. In the end, a multi-layer perceptron was trained to classify images into two classes: melanoma and benign. The experimental assessments showed that our proposed model attained the best detection accuracy compared to existing techniques. The main contributions of the proposed technique are given below.

- To propose a novel features fusion-based technique for the early detection of skin cancer. First, images were pre-processed using GF to remove the noise. Second, we extracted features from the images using LBP and Inception V3. Then, we fused these features and employed an LSTM network for the binary classification into malignant and benign. Additionally, we used an Adam optimizer to adjust the learning rate for Inception V3.
- Our proposed model is an efficient technique due to its hybrid architecture that extracts most representative features and employs Long Short-Term Memory (LSTM) for the classification.
- We trained our classifier on 75% dataset and performed various experiments for the assessment of the proposed system, demonstrating its efficacy
- We cross-validated our proposed model, and the experiments showed that it significantly outperformed the existing techniques.
- Our proposed features fusion-based model is simple and easy to execute.

The remaining paper is organized as follows: Section 2 presents related work, Section 3 explains our proposed method, Section 4 demonstrates various experiments, and Section 5 is the conclusion.

## 2. Related Work

Initially, traditional machine learning-based techniques were introduced for melanoma detection and classification. Codella et al. developed a traditional ML-based features extraction method employing the color and edge histogram along with local binary pattern (LBP). The shades of gray algorithm is used for image pre-processing in [1]. Moreover, Mask R-CNN, a deep learning approach, is used for the segmentation of skin lesions. Morphological operations were performed to remove noise in the images. The detection of skin lesions from dermoscopy images consisting of three steps was performed in [22], such as: (1) image pre-processing to improve the performance of the model dividing them into negative and positive classes, (2) image augmentation applied on data to protect the model from overfitting, (3) the use of Densnet-121 to extract the features and the proposal of a U-net architecture-based lightweight CNN model for skin lesion detection. The results of the proposed method are not mentioned in terms of confusion matrix and accuracy.

H. A. Hasan proposed a framework, i.e., hybrid detection techniques for skin cancer detection, in which images were converted from RGB to grayscale. The list of arrays was converted into NumPy array, and benign images were labeled as 0 and malignant images as 1. The dataset was divided into train and test samples, and the CNN model was trained with K-fold validation. The Xception model achieved 85.303% accuracy [23], whereas other tested models such as MobileNet-v2, Resnet 50, and VGG19 achieved lower accuracy, with MobileNet-v2 producing the lowest at 54.54%. The lower accuracy was due to using a dataset with low-quality images. In [24], skin cancer detection was performed by employing a machine learning model, i.e., Support Vector Machine (SVM). Features were extracted by the gray level co-occurrence matrix (GLCM) method and fed to SVM for the detection of skin lesions. The method achieved 95% accuracy; however, it could be improved by utilizing some image pre-processing methods on the dataset to remove noise and improve the training process.

In [25], M. R. Ibraheem performed the contrast-limited adaptive histogram equalization (CLAHE) technique to enhance the images of lesions, employing bilinear interpolation and threshold equalization methods. Moreover, the pixel-based method was applied for the segmentation of lesions and feature extraction. The classes were named as 0 to 2, where 0 represented a background object, 1 indicated the benign lesion, and 2 represented melanoma. Gradient Boosted Tree (GBT) gave 97.5% accuracy. In [26], Rahajeng, M. Nuh used different techniques, such as median filter, threshold and automatic cropping as image pre-processing operations. Furthermore, active contour and Sobel filters were applied for skin lesion segmentation based on shape, color, and texture features, which were obtained using the GLCM method. SVM was employed as a classifier to identify the type of skin lesion, achieving 85% accuracy. The performance of the system was lacking in high precision and accuracy.

G. S. Jayalakshmi and V. S. Kumar initially resized images into 400 × 400 pixels and fed these into an image input layer. A convolution layer with 32 filters was used for feature extraction, changing the parameter of input distribution, and adaptive re-parameterization was applied using a batch normalization layer to overcome the internal covariant shift [27]. ReLU was used as an activation function after max-pooling and fully connected layers. Accuracy, recall, F1-score, and precision were calculated as performance metrics. The learning rate was set as 0.001, and 89.3% accuracy with a loss factor of 0.2633 was attained; however, the accuracy can be improved by improving the training options and customizing the layers of the proposed method. The authors of [28] used CNN to classify an image as benign or malignant. The International Skin Collaboration (ISIC) 2016 dataset was used for training, which consisted of images with dimensions of 1024 × 767 pixels. These images comprised three types, i.e., melanoma was classified as malignant, whereas nevus and seborrhea keratosis were classified as benign. The generalized Gaussian distribution method was used for image segmentation with a convolution neural network employing ReLU as an activation function to classify the images. The performance of the model was calculated in terms of accuracy, specificity, and sensitivity. The accuracy of the proposed method was 98.32%; however, the model is not validated on other datasets.

Y. Filali, H. El Khoukhi focused on classification of a skin lesion by decomposing the images into object component and texture. Segmentation was applied on objects to obtain the desired area; then, the segmented area and texture were combined. A convolutional layer was used for feature extraction to learn the hidden patterns [29] requiring high computational resources. A pooling layer was used after the convolution layer to reduce the spatial size, and fully connected layers were added to use the extracted features for classification of the skin lesion. Softmax was used as an activation function to classify melanoma as malignant and nevus as benign. The classification results were evaluated through accuracy, specificity, and sensitivity. The proposed method achieved an accuracy of 93.50%, which can further be increased. N. Rezaoana classified nine classes—NV, DF, MEL, VASC, BCC, AKIEC, BKL, squamous carcinoma, and seborrhea keratosis—using the CNN model. Data augmentation on images such as flip, rotation, shear, etc., were performed to increase the volume of data for training. Convolutional layers were used for feature extraction, the max pooling layer was utilized to reduce the dimensionality, and softmax was used after the pair of fully connected layers as an activation function [30]. Precision, F1-score, recall, and accuracy were used as the performance measurements of the model. VGG-16 and VGG-19 were also analyzed and achieved an accuracy of 69.57% and 71.19%, respectively, whereas the proposed model achieved an accuracy of about 79.45%; however, this accuracy could be increased by improving the model architecture. Various existing techniques are reported in Table 1.

## 3. Materials and Methods

**Figure 1:**
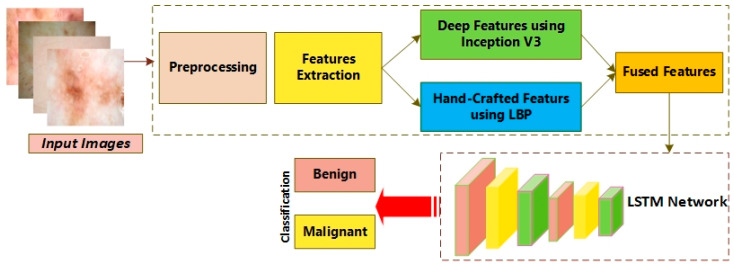
Architecture of the proposed skin cancer detector.

In Figure 1, the workflow of the proposed system is presented. The figure demonstrates that the proposed model consisted of pre-processing utilizing the Gaussian Filtering method to minimize the noise present in the images. Then, features extraction was performed using LBP and the deep learning model, i.e., the Inception V3 algorithm. Moreover, an Adam optimizer was exploited to optimize the rate of learning for the Inception technique. Ultimately, Long Short-Term Memory (LSTM) was utilized to detect and classify the skin images into melanoma and benign.

### 3.1. Gaussian Filtering:

To improve the images and remove the noise, 2D Gaussian filters were employed. These required high computational resources; however, they provide a new area to conduct research. In this sub-stage, Gaussian operators represent convolutional operators, and smoothing is recommended by convolution. The one-dimensional Gaussian operator is given below as:

(1) $$G_{1d}{}^{\left(x\right)}=\frac{1}{\sqrt{2\pi ϭ}}e^{-\left(\frac{x^{2}}{2{ϭ}^{2}}\right)},$$

The significant filter for the smoothing of images passes through localization in the frequency and spatial domains, whereas the relation of uncertainty is executed as shown below:

(2) $$\Delta x\Delta ɯ\;\geq \;\frac{1}{2},$$

Two-dimensional operator for Gaussian filter is given below:

(3) $$G_{2d}\left(x,y\right)=\frac{1}{2\pi {ϭ}^{2}}e^{-\left(\frac{x^{2}+y^{2}}{2{ϭ}^{2}}\right)}$$

Here, sigma (ϭ) is the standard deviation for a Gaussian function. If the value is maximum, the smoothing will be highest, whereas *x* and *y* exhibit the Cartesian coordinates of the image, demonstrating the window dimensions.

### 3.2. Features Extraction (FE)

Features extraction was performed after pre-processing the data using the fusion of algorithms, i.e., local binary patterns (LBP) and a histogram of orientation gradients (HOG) with the Inception v3 method. Moreover, we utilized the learning rate scheduler along with the Adam optimizer to improve the detection performance.

### 3.3. Local Binary Patterns:

LBP has been utilized in various domains, such as knee disease detection [40], eye disease detection [41], etc. As a separate vector, the histograms were integrated in LBP, namely as a pattern vector. The LBP texture features were integrated along with a self-organizing map (SOM), which finds an alternative use in assessing the efficacy of the proposed technique. LBP is a method for texture description depending upon the differential symptoms from central and neighboring pixels. A binary code was attained by employing the threshold technique on all pixel values utilizing the mid pixel. The binary code is known as the binary pattern. The value of the neighbor pixel was set as 1 where the value of the pixel became greater than the value of the threshold. This was set as 0, where the value of the pixel was less than the value of the threshold. Succeeding this, a histogram was utilized to compute the occurrence of BP, and each pattern exhibited the possible value for the pattern in an image.

The LBP module uses the intermediate pixel’s value as a threshold to the neighboring 3 × 3 pixel. The threshold was employed by deploying a binary pattern that referred to texture features. The LBP procedure is defined in equation form below:

(4) $$LBP_{\left(v_{c},u_{c}\right)}=\overset{7}{\underset{i=0}{\sum }}2^{i}g(I_{i}-I(v_{c},u_{c})),$$

$LBP_{\left(v_{c},u_{c}\right)}$ exhibits the LBP value for the middle pixel ($v_{c},u_{c})$. $I(v_{c},u_{c})$ and $I_{i}$ represents the central and neighboring pixel values, whereas index *i* demonstrates the index of the neighbor pixel as for *v* < 0 the *g*(*v*) = 0 and *g*(*v*) = 1 for *v ≥* 0. Consequently, the nearest pixels may be 0, if the value of the score is less than the threshold. On the other side, it may be set to 1, where the value of the pixel is greater than the value of the threshold. LBP is computed through scalar multiplication among weight and binary matrices. In the end, the results are used to exhibit the value of LBP.

### 3.4. Inception V3 Using Adam Optimization

Convolutional neural networks (CNN) consist of five layers: the input layer, convolution layer, pooling layer, fully connected layer, and classification layer, which is known as the output layer. Google Net is a network installed in Google. It utilizes the Inception model as it bounds the attributes of layers and improves the depth. Consequently, it has been widely used for classification purposes. The general attributes of CNN were explained in [42].

Convolutional Layer: Convolutional layers may have variations when all pixels are not linked to previous layers through parameters and bias. The image is split into small regions and then parameters and biases are utilized. These biases and parameters are known as filters which are convolved with the tiny regions on the input image and provide feature maps. The filters are known as simple features that may be analyzed in images fed into the input layer, in that specific layer. Moreover, the number of parameters is important during convolutional operation and can be small, as the same filter is employed for a complete image against a single feature. The hyper-parameters of a convolutional layer include the size of the local region, the number of filters, padding, and stride. To attain optimal outcomes, the hyper-parameters are fine-tuned depending upon the size of the input image.

Pooling Layer (PL): PL is utilized to minimize the spatial dimensions of images, the number of parameters, and the complexity. It employs a constant method for an input without parameters. There are various types of PL, such as max, average, and stochastic pooling. The most common type is max pooling, which is applied when *i* x *i* slides through and reduces the input, according to stride *‘s’*. The size of the input becomes limited and the maximum value in the *i* × *i* region is utilized. It provides translational invariance when a tiny difference in a position is used to assess an input image. Therefore, the location disappears while minimizing the size.

Fully Connected Layer: In this layer, the result from the final PL is provided as input. It works as a convolutional neural network with all neurons connected to the present layer. Therefore, the convolutional layer contains the maximum number of parameters. Then, the fully connected layer is attached to the final layer, which is known as the classification layer.

Activation Function (AF): Various AFs are employed for various types of CNN. The optimal output is attained through non-linear activation functions than tangent or sigmoid functions. These functions are employed to increase the speed of training. Therefore, different activation functions are employed, whereas ReLU demonstrates better performance than other models. Convolutional neural network (CNN) utilizes vector computation and the chain rule. Let us suppose that x is a scalar as x ꞓ R, and y ꞓ Rh as a vector, where x is a function of y, representing the partial derivative of x in the context of y, which is mathematically described as:

(5) $$\left(\begin{array}{c} \partial x\\ \partial Y \end{array}\right)=\frac{\partial x}{\partial Y_{i}}$$

More specifically, $\left(\begin{array}{c} \partial x\\ \partial Y \end{array}\right)$ represents the vector comprising an equal size to *Y,* and the *ith* number component is represented by $\left(\begin{array}{c} \partial x\\ \partial Y \end{array}\right){\;}_{i}$. It is noteworthy that $\left(\begin{array}{c} \partial x\\ \partial Y^{t} \end{array}\right)=\left(\begin{array}{c} \partial x\\ \partial Y \end{array}\right){\;}^{t}$. Additionally, *z ꞓ R^w^* represents a different vector, whereas *Y* is a function of *z.* Moreover, the fractional derivative of *Y* in terms of *z* is represented by:

(6) $${\left(\begin{array}{c} \partial x\\ \partial Y^{t} \end{array}\right)}_{ij}=\frac{\partial Y}{\partial z_{i}}$$

In the fractional derivative *h* × *w* matrix, it is accessed at the interval of the i and j row and column, respectively, such as (∂*Y_i*)/(*∂z_i*). It is clearly shown that *x* is a task of *z* in chain arguments. Thus, one method maps *z* to *Y*, while another method maps *Y* to *x*. The chain method was employed for the computation presented below.

(7) $$\left(\begin{array}{c} \partial x\\ \partial z^{t} \end{array}\right),\;\mathrm{as}\;\left(\begin{array}{c} \partial x\\ \partial z^{t} \end{array}\right)=\left(\begin{array}{c} \partial x\\ \partial Y^{t} \end{array}\right)\;\left(\begin{array}{c} \partial x\\ \partial z^{t} \end{array}\right)\;,$$

The loss computation function is used to compute the difference among the predicted value of the Convolutional Neural Network *x^l^* and the goal *g, z*^1^*→w*^1^*, z*^2^*→,……,z^l^→w^l^ = x.* The loss function is simple, as *x = ||target-z^l^||*^2^. The predicted output is represented as argmax*_i_ z_i_^l^.* Therefore, a convolutional operation is computed as below:

(8) $$Y_{i}^{l+1},j^{l+1},d=\overset{h}{\underset{i=0}{\sum }}\overset{w}{\underset{j=0}{\sum }}\overset{D}{\underset{d=0}{\sum }}f_{ijd\;}\times \;z_{i^{l+1}+,\;i,j^{l+1},d}^{l}$$

where *f* represents the filter with the size (*h* × *w* × *d^l^*). Therefore, the conv. layer maintains the size *(h^l^-h +* 1) × *(w^l^ – w +* 1), comprising *d* slices that represent *Y(z^l+1^)* in $R^{h^{l+1\;}\times \;w^{l+1\;}\times \;w^{l+1\;}\;},$ *h^l+1^ = h^l^ − h +* 1, *w*^l +^ ^1^ *= w^l^ − w+*1, and *d* ^l + 1^ = *d*.

The likelihood of all labels *k* ꞓ {1,…,*k*} is employed for the training of instance that is computed through *P(k|z)* = $\frac{\exp\left(x_{k}\right)}{\sum_{i}^{k}\exp(x_{i})}$; here, *x* refers to the non-normalized log possibility. The ground truth is *s(k|z)*, which is normalized in a way that $\underset{k}{\sum }s\left(k|z\right)=1$, where loss is computed using cross-entropy and is represented as below:

(9) $$l=\overset{n}{\underset{k=1}{\sum }}\log\left(p\left(k\right)\right)s\left(k\right),$$

The value of cross-entropy is based on differentiation in the context of *x_k_*, and it is computed as the gradient training of deep functions as it has the easiest form $\frac{\partial l}{\partial x_{k}}=p\left(k\right)-s\left(k\right)$, which varies from −1 to 1. The cross-entropy decreases due to the possibility of a maximum value of probability for an accurate label. Inception version 3 is referred to as mutual for the labels that are free of trained instances *v(k)* with a parameter € representing a training sample; then, the share label *s(k|z)* is easily returned as:

(10) $$s^{\prime (k|z)}=\left(1-\varepsilon \right)\delta_{k,z}+\frac{\varepsilon }{K}$$

In another case, the cross-entropy is computed as below:

(11) $$h\left(s^{\prime },p\right)=-\overset{K}{\underset{k=1}{\sum }}\log\left(p\left(k\right)\right)s^{\prime \left(k\right)}=\left(1-\varepsilon \right)h\left(q^{\prime },p\right)+\varepsilon h\left(v,p\right),$$

Therefore, the regularization for label smoothing is similar for the implication of cross-entropy loss *h(s,p)* and losses as *h(s,p)* and *h(v,p).*

**Figure 2:**
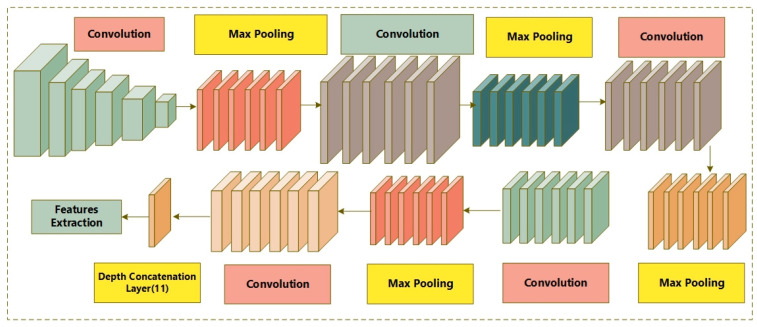
Architecture of Inception V3.

**Figure 3:**
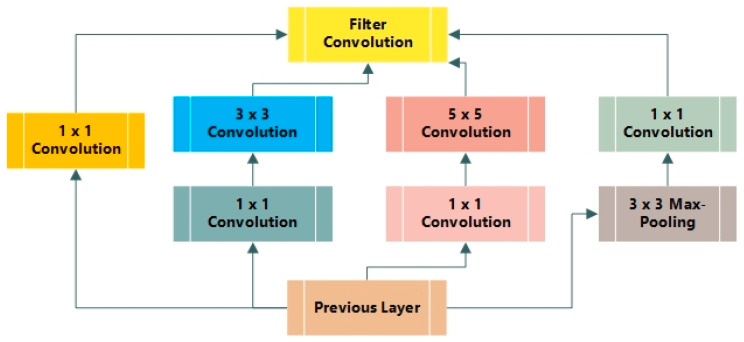
Structure of basic Inception model.

Google Net is known as an Inception network due to its aim of acting similarly [43]. It comprises various versions, such as V1, V2, V3, V4, and Incep-ResNet. Moreover, the Inception network consists of three varying sizes of max-pooling and convolutional layers. Various channels pass through the network layers and, after convolutional operation, the non-linear fusion method is employed. In Figure 2, the basic architecture of the Inception network is shown. Version 3 of the Inception network employed by Keras is pre-trained on ImageNet. The size required for images is 299 × 299 with three channels. Comparatively, Inception V3 utilizes a convolutional kernel method to split integrals into the tiniest convolution. In any instance, 3 × 3 conv. is split into 1 × 3 and 3 × 1 convolutions. The number of attributes is limited; therefore, the network speed is enhanced while effectively extracting spatial features. The three different grid sizes are 8 × 8, 17 × 17, and 35 × 35. The structure of the basic Inception model is presented in Figure 3.

### 3.5. Learning Rate Scheduler

During the deep learning training stage, limiting the learning rate $\nabla_{t}$ is suggested when the training accuracy increases. The number of parameters is improved during training, which is referred to as the learning rate (LR) or step size. More particularly, LR adjusts the hyper-parameter, which is employed to train the neural network, utilizing values from 0 to 1. The minimum values for the learning rate require more epochs for training, and the weights alterations are reduced. On the other hand, the more variations there are in LR, the less training epochs that are required by the neural network. The maximum LR creates a divergent training procedure, whereas minimum LR causes a slow convergence process. The procedure used to schedule LR is known as learning rate scheduling. There are various general LR schedulers, such as step, time, and exponential decay. An Adam optimizer is an estimator for moments that employ a method based on first-order gradient. It also relates to the adaptive prediction of low-order moments. Where $g_{T}$ refers to gradients, $\vartheta_{T}$ represents the weight at time *T,* $\beta_{i}\;$ and $\beta_{j}$ are 0 and 1, and *℺* represents LR. $g_{T}^{2}$ represents the square of $g_{T}\times g_{T}$. The initial settings were *℺* = 0.001, $\beta_{i}$ = 0.98, $\beta_{j}$ = 0.999, and ꞓ = $10^{-7}$. 

### 3.6. Fusion Process

Features fusion is utilized in various machine learning and computer vision applications, such as medical imaging [40,41]. It provides a vital process that incorporates most of the features’ maps. The proposed method is based on entropy for features fusion. Additionally, the attained features are merged to form a single vector. We computed three vectors here, as below:

(12) $$f_{IncepV3\;\times \;m}=\left\{IncepV3_{1\times 1},IncepV3_{1\times 2},IncepV3_{1\times 3},IncepV3_{1\mathrm{xn}}\right\},$$

(13) $$f_{LBP1\;\times \;p}=\left\{LBP_{1\times 1},LBP_{1\times 2},LBP_{1\times 3},\ldots \ldots ,LBP_{1\mathrm{xn}}\right\}$$

Features fusion was applied as below:

(14) $$Fusion{\left(Feature_{vector}\right)}_{1\;\times \;s}=\overset{2}{\underset{i=1}{\sum }}\{fInceptionV3_{1\;\times \;m},\;fLBP_{1\;\times \;p}\},$$

Here, *f* denotes a fused feature vector. Afterwards, an entropy is computed for selective features based on the value, as shown below.

(15) $$L_{he}=-Nhe_{b}\overset{n}{\underset{i=1}{\sum }}p\left(f_{i}\right),\;$$

(16) $$F_{sel}=L_{he}\left(\max\left(f_{i},1186\right)\right),$$

Here, *p* presents the probability of features and $L_{he}$ refers to an entropy. In the end, the selected features are fed to the classification network to distinguish the affected images.

### 3.7. Classification Using LSTM

A convolutional neural network is one of the most popular artificial neural networks (ANN) that performs mathematical linear operations on feature vectors known as convolutional [44]. CNN operates in two phases, i.e., the forward phase and the back propagation phase during training. Input and weights are multiplied with a matrix of the filter; then, a convolutional operation is performed to calculate the output, and this output is used for error computation in the forwarding phase. The parameters are adjusted during the back propagation phase to overcome the final prediction errors. Ground truth and output are compared to find errors using the cost function [45]. The gradient of the parameter is computed, and parameters are updated to minimize the error.

There are multiple layers in convolutional neural networks, such as the convolutional layer, pooling layer, normalization layer, fully connected layer, activation layer, and classification layer. CNN performs significantly in the problems related to image data. The layers’ detail is shown in Table 2.

## 4. Experimental Evaluation

In this section, we discuss the environmental setup, the metrics employed for the assessment, and the various experiments used to analyze the performance of the proposed model. 

### 4.1. Dataset

**Figure 4:**
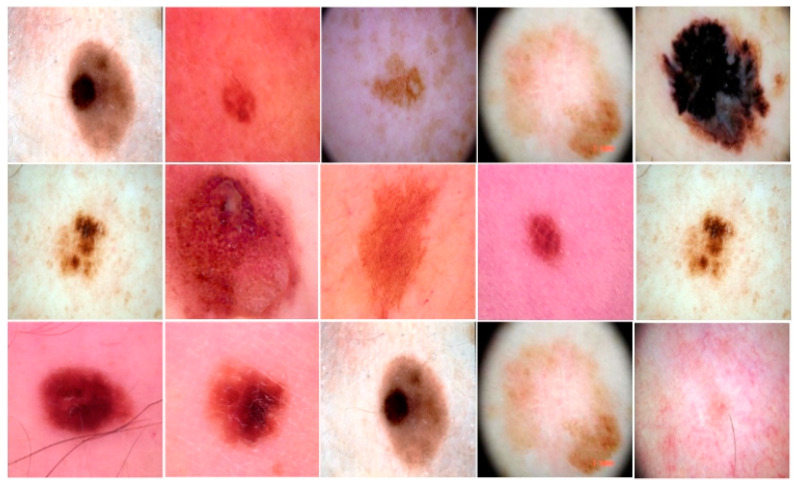
Some samples from the dataset.

**Figure 5:**
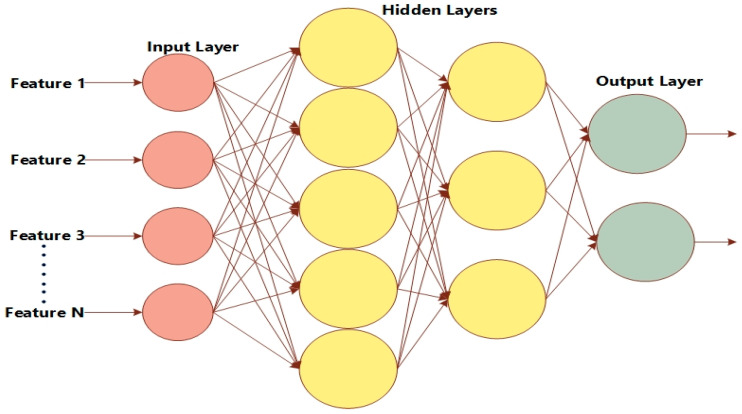
The structure of LSTM.

The dataset was collected from the skin lesion DermIS dataset, which is available on the Kaggle website [46]. There are two classes in the dataset: benign and malignant. Seventy-five percent of the images of each class were used to train the model, and the remaining 25% were used for validation purposes. There were 1000 images in total; 500 images belonged to the benign class and 500 to the malignant class, with dimensions of 600 × 450 pixels. Image pre-processing was performed to resize the images to dimensions of 227 × 227, and the resized images were provided as input to the image input layer. The images of the benign and malignant classes used to train our model are provided in Figure 4. The employed LSTM classifier’s approach is shown in Figure 5.

### 4.2. Metrics

To assess the proposed model, we utilized various metrics, such as precision, recall, accuracy, and F1-score. These metrics relied on true-positive (*TP*), false-positive (*FP*), true-negative (*TN*), and false-negative (*FN*). *TP* refers to the number of correctly classified images by our proposed model; *FP* refers to the number of images that were incorrectly classified as other malignant images; *FN* denotes the number of diseased images that were incorrectly classified as normal; and *TN* refers to the number of images that were correctly classified as a negative class, such as normal or benign. Furthermore, precision refers to the fraction of *TP* over the total images classified as positive. The mathematical equation is given below.

(17) $$Precision=\frac{TP}{TP+FP}\;$$

The accuracy of the system indicates the correctly classified images by the proposed system. The equation is presented below.

(18) $$Accuracy=\frac{TP+TN}{TP+TN+FP+FN}$$

The recall is the fraction of the classified positive class images to all images of the positive class, whether they were classified as a negative class by the system. The recall value closer to 1 refers to the better model. The recall equation is given below.

(19) $$Recall=\frac{TP}{TP+FN}$$

Another metric used for the proposed system was the F1-score. This is defined as a measure of the accuracy of the proposed model over the dataset. It is employed for binary classification models. The equation of the F1-score is given below.

(20) $$F1\;score=2*\;\frac{Precision*Recall}{Precision+Recall}$$

### 4.3. Environmental Setup

We performed the experiments using a GPU NVIDIA card, i.e., GEFORCE GTX with 4 GB memory. The details of the employed hardware are shown in Table 3. The operating system was Windows 10 with a 16 GB RAM. The experiment was performed on the Anaconda framework.

### 4.4. Results

**Figure 6:**
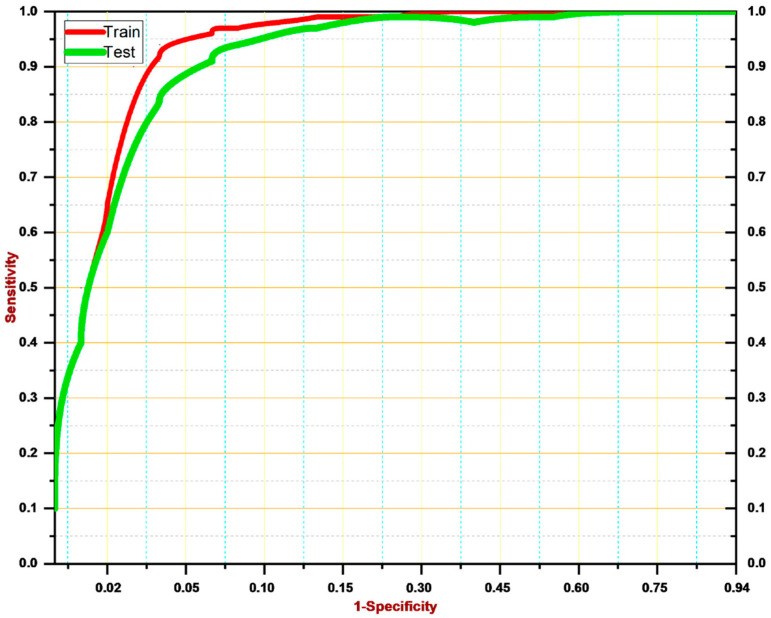
ROC curve for the proposed skin cancer detector.

In this section, we discuss the results achieved by our proposed model during the testing phase. As shown in Figure 6, the ROC curve exhibits the significant performance of the proposed model. We attained 99.4% accuracy, 98.7% precision, 98.66% recall, and a 98% F-score. More specifically, we employed 750 images to train the proposed technique, including 375 images from the malignant class and 375 images from the benign class. As we employed transfer learning-based algorithms, we achieved considerable results while only using a small number of training samples. Then, we tested our proposed model over the remaining 30% of test data, i.e., 250 images belonging to the malignant and benign classes, respectively. We achieved 99.4% accuracy over the testing data, which demonstrates the tremendous performance of the proposed system. The number of TPs were 123, and TNs were also 123 by our proposed system. In particular, we believe that our proposed model has a significant ability to detect skin cancer in images and classify it into the malignant class. Furthermore, it also classifies non-affected images as benign images.

### 4.5. Comparison with Segmentation-Based Methods

**Figure 7:**
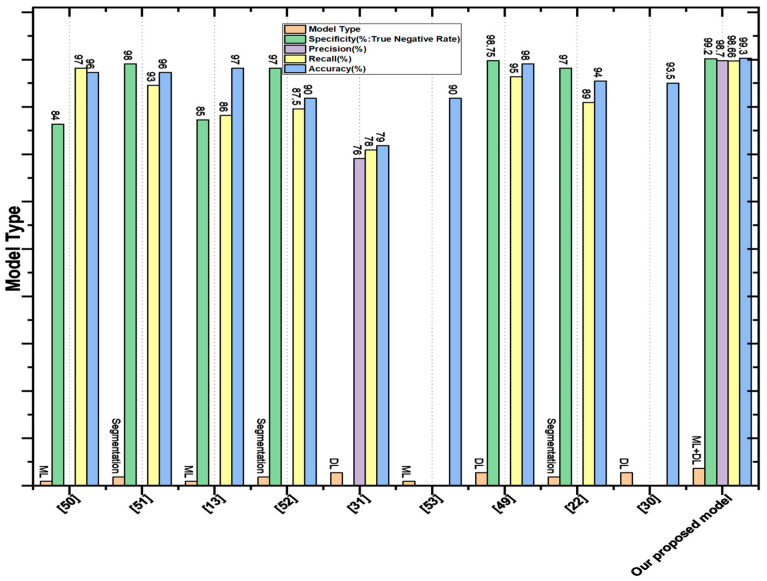
Comparison plot with existing techniques.

The aim of [47] was the detection of skin cancer from images using segmentation and feature extraction methods. Image pre-processing was employed to improve the quality of images, and a median filter was applied to reduce the noise. A median filter of a 5 × 5 windows size was used after morphological operations erosion, and dilation was performed to remove extra artifacts such as hair and skin color. Binary mask and threshold techniques were applied to obtain the desired area of the image. The asymmetry, border irregularity, color, and diameter of the area were selected for the detection of skin cancer. The proposed method detected skin cancer with 90.83% accuracy. Manu Gofal [48] performed lesion boundary segmentation from dermoscopic images with the deep learning method. The PH2 and ISIC 2017 datasets were used for the segmentation of skin lesions, and image pre-processing was performed to reduce the computational cost and improve performance. The performance measures were sensitivity, specificity, and accuracy, achieving: 98%, 92%, and 93% respectively. The goal of [49] was the automatic melanoma class segmentation of skin cancer using deep learning. The U-Net architecture of the convolutional neural network was applied for better segmentation of skin lesions. Holes, disconnected regions, and loose objects were still present after U-Net segmentation; therefore, post-processing was performed by applying morphological operations dilation. Noise and extra artifacts such as hair and skin color were removed from images for better segmentation. The proposed methodology achieved 96% accuracy, 98% specificity, and 93% sensitivity, whereas our proposed model attained 99.4% accuracy, 98.7% precision, 98.66% recall, and a 98% F-score. A comparison of the proposed CNN with existing segmentation-based and other techniques is presented in Figure 7. The figure clearly shows that our proposed model achieves remarkable results compared to existing segmentation-based techniques.

### 4.6. Comparison with DL-Based Methods

In this section, we compare our proposed features fusion method for skin cancer detection with existing DL-based methods. N. Rezaoana classified nine classes—NV, DF, MEL, VASC, BCC, AKIEC, BKL, squamous carcinoma, and seborrhea keratosis—using the CNN model. Data augmentation on images, such as flip, rotation, shear, etc., were performed to increase the volume of data for training. Convolutional layers were used for feature extraction, a max pooling layer was utilized to reduce the dimensionality, and softmax was employed after pairing of the fully connected layer as an activation function [30]. Precision, F1-score, recall, and accuracy were used as performance measurements of the model. VGG-16 and VGG-19 were also analyzed and achieved an accuracy of 69.57% and 71.19%, respectively, whereas the proposed model achieved an accuracy of about 79.45%. The objective of [50] was the detection of skin lesions from skin images using an artificial neural network. The PH2 dataset was utilized, containing 40 images from the melanoma class and 160 images from the benign class. Image pre-processing was performed for the removal of noise and accurate segmentation. Moreover, area, shape, and centroid features were extracted from the images, and these features were fed to the artificial neural network for the detection of skin cancer, attaining 98% accuracy. Y. Filali and H. El Khoukhi focused on the classification of a skin lesion by decomposing the images into object components and texture. Segmentation was applied to objects to obtain the desired area, then the segmented area and texture were combined. A convolutional layer was used for feature extraction to learn the hidden patterns [29]. A pooling layer was used after the convolution layer to reduce the spatial size. Furthermore, fully connected layers were added to use the extracted features for the classification of skin lesions. Softmax was employed as an activation function to classify melanoma as malignant and nevus as benign. The classification results were evaluated through accuracy, specificity, and sensitivity. The proposed method achieved an accuracy of 93.50%. A comparison of accuracy with the existing DL-based and ML-based models is shown in Table 4. The plot of the comparison is shown in Figure 7.

### 4.7. Cross-Validation

**Figure 8:**
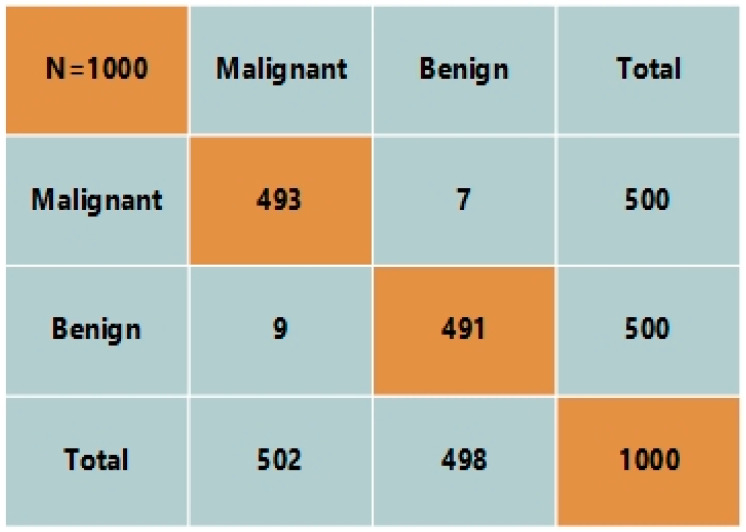
Confusion matrix for cross-validation.

In this section, we performed cross-validation on our proposed system for skin lesion detection. We employed a different dataset from Kaggle for this purpose, namely International Skin Image Collection (ISIC). The dataset consists of 1800 images from the malignant class and 1497 images from the benign class. We utilized 500 images from each class to analyze the performance. Our proposed techniques classified 493 images from the malignant class and 491 images from the benign class. The confusion matrix is shown in Figure 8. The figure clearly shows that our proposed model achieved 98.4% accuracy over cross-validation. The results show that our proposed model is a robust technique that can detect skin cancer in images at an early stage and with high precision. Therefore, it is concluded that our proposed model outperforms the existing methods for skin disease detection.

### 4.8. Discussion

The above-mentioned experimental results indicate that our proposed skin lesion detector is a more effective system than existing techniques. In [53], the authors employed the fast random forest (FRF) algorithm to identify the area affected by malignant melanoma. They achieved 17% precision discordance with the pathologist’s results. Contrastingly, in [54], the swarm optimization technique was utilized to extract the region of interest from the dermoscopy images. Then, speeded-up robust features were extracted and images were classified by employing CNN. The classification accuracy was 98.42% and the precision was 97.73%. Both methods achieved significant results; however, they failed in the case of unseen tiny skin lesions. Moreover, our proposed model attained 99.4% detection accuracy and 5% discordance with the pathologist’s results. We believe that the hybrid nature of our proposed system for feature extraction—i.e., ML and DL-based methods—make our system capable of extracting the most representative features from skin lesions. We used an Adam optimizer to adjust the learning rate that optimized the training phase of the proposed classifier. Furthermore, the LSTM classifier reduces the complexity by minimizing the requirement to update the weights rather than convolution-based neural networks. Thus, our proposed model is an efficient skin lesion detector achieving 98.7% precision, 98.66% recall, and a 98% F-score. The proposed system identifies skin lesions with significant accuracy and outperforms the existing skin lesion detection methods.

## 5. Conclusions

In this study, a novel and robust skin cancer detection model was proposed based on features fusion. In the first stage, our proposed model pre-processed the images using a GF filter to remove the noise from the skin images. Then, features were extracted by employing LBP for manual features extraction and Inception V3 for automatic features extraction. Aside from this, an Adam optimizer was utilized for the adjustments of the learning rate. In the end, an LSTM network was utilized on fused features for the classification of skin cancer into two classes: malignant and benign. We utilized the skin lesion DermIS dataset available on the Kaggle website, consisting of 1000 images, out of which 500 belong to the benign class and 500 to the malignant class. The proposed methodology attained 99.4% accuracy, 98.7% precision, 98.66% recall, and a 98% F-score. We evaluated our proposed model and compared the performance with existing segmentation-based and DL-based techniques. The results show that our method provided significant results compared to existing techniques. In the future, we aim to employ our proposed model for multi-classification for skin cancer detection, such as squamous cell carcinoma, basal cell carcinoma, Kaposi’s sarcoma, Merkel cell carcinoma, etc.

## Figures and Tables

**Table 1:** Summary of some existing techniques with their challenges.

Ref.	Year	Dataset Used	Classes of Skin Lesion	Activation Function Used	Model	Model Type	Accuracy (%)	Issues
[31]	2013	176 dermoscopy images	Binary Classification	-	Gradient Histogram, and BOF	Supervised	96	Generalization problem
[32]	2020	ISIC 2019	NV, DF, MEL, VASC, BCC, AKIEC, BKL	RELU	CNN	Supervised	96	Low-level features are not considered
[33]	2020	ISIC	Benign, malignant	-	SVM, KNN, and CNN (Hybrid)	Supervised	KNN:57.3 SVM:71.8	Less detection accuracy
[34]	2019	ISIC 2017, PH2	Melanoma, non-melanoma	RELU	CNN	Supervised	95	Low-level features are not considered
[35]	2021	HAM10000	Benign, malignant	SIGMOID	CNN	Supervised	90.93	Less detection accuracy
[36]	2020	ISIC2018, HAM10000	Melanoma, nevus, seborrheic keratosis	SOFTMAX	CNN	Supervised	86	Less detection accuracy
[23]	2020	ISIC	Benign, malignant	RELU	CNN	Supervised	80	Less detection accuracy
[37]	2020	PH2	Melanoma, atypical nevus, common nevus	SOFTMAX	CNN	Supervised	95.0	Overfitting issue
[38]	2020	HAM1000	NV, DF, MEL, VASC, BCC, AKIEC, BKL	RELU	CNN	Supervised	90	Low precision and accuracy
[39]	2019	SLC 2017, ISBI 2016, and PH2	Melanoma, non-melanoma	RELU	ResFCN	Supervised	94.29	High computational resources

**Table 2:** Details of the proposed LSTM classifier.

Type	Learnable	Activation
Feature input	-	7
LSTM-1	Input weights 512 × 7 Recurrent weights 512 × 128 Bias 512 × 1	128
5 × [Batch Normalization]	Offset 128 × 1 Scale 128 × 1	128
8 × [RELU]	-	128
addition	-	128
7 × [LSTM-2]	Input weights 512 × 128 Recurrent weights 512 × 128 Bias 512 × 1	128
Fc_1	Weights 22 × 128 Bias 22 × 1	22
Fc_2	Weights 22 × 22 Bias 22 × 1	22
SOFTMAX	-	22
Class output	-	22

**Table 3:** System Specifications of the proposed method.

Hardware	Specifications
Computer	GPU Server
CPU	Intel Core i5
RAM	16 GB
GPU	NVIDIA GEFORCE GTX × 4

**Table 4:** Comparison with existing techniques.

Ref	Model Type	Specificity (% True Negative Rate)	Precision (%)	Recall (%)	Accuracy (%)
[51]	ML	84	-	97	96
[49]	Segmentation	98		93	96
[13]	ML	85	-	86	97
[47]	Segmentation	97	-	87.5	90
[30]	DL	-	76	78	79
[52]	ML	-	-	-	90
[50]	DL	98.75	-	95	98
[48]	Segmentation	97	-	89	94
[29]	DL	-	-	-	93.5%
Our proposed model	ML+DL	99.2	98.7	98.66	99.4

## Data Availability

The data used for this study are publicly available. Further, any queries related to the dataset may be directed to the authors.
